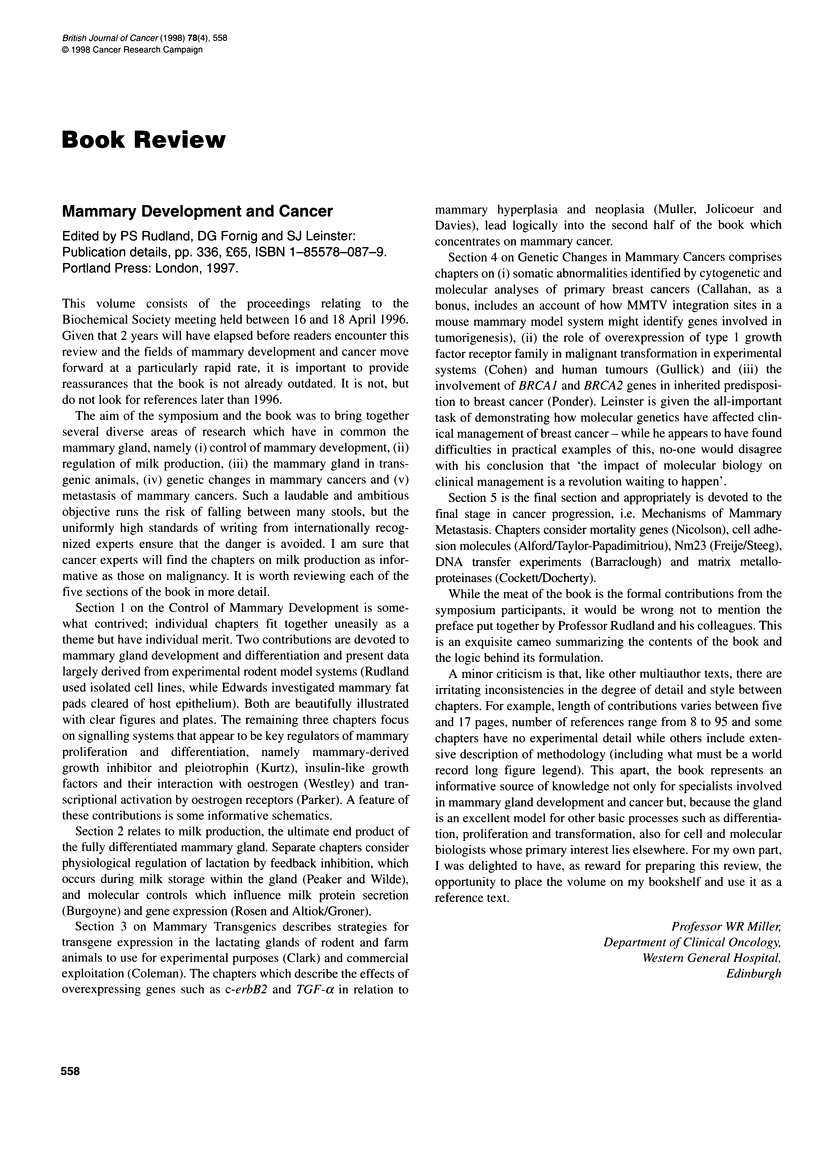# Mammary Development and Cancer

**Published:** 1998-08

**Authors:** WR Miller


British Journal of Cancer (1998) 78(4), 558
© 1998 Cancer Research Campaign

### Book Review

### Mammary Development and Cancer

Edited by PS Rudland, DG Fornig and SJ Leinster: Publication details, pp. 336, £65, ISBN 1–85578–087–9. Portland Press: London, 1997.

This volume consists of the proceedings relating to the Biochemical Society meeting held between 16 and 18 April 1996. Given that 2 years will have elapsed before readers encounter this review and the fields of mammary development and cancer move forward at a particularly rapid rate, it is important to provide reassurances that the book is not already outdated. It is not, but do not look for references later than 1996.

The aim of the symposium and the book was to bring together several diverse areas of research which have in common the mammary gland, namely (i) control of mammary development, (ii) regulation of milk production, (iii) the mammary gland in transgenic animals, (iv) genetic changes in mammary cancers and (v) metastasis of mammary cancers. Such a laudable and ambitious objective runs the risk of falling between many stools, but the uniformly high standards of writing from internationally recognized experts ensure that the danger is avoided. I am sure that cancer experts will find the chapters on milk production as informative as those on malignancy. It is worth reviewing each of the five sections of the book in more detail.

Section 1 on the Control of Mammary Development is somewhat contrived; individual chapters fit together uneasily as a theme but have individual merit. Two contributions are devoted to mammary gland development and differentiation and present data largely derived from experimental rodent model systems (Rudland used isolated cell lines, while Edwards investigated mammary fat pads cleared of host epithelium). Both are beautifully illustrated with clear figures and plates. The remaining three chapters focus on signalling systems that appear to be key regulators of mammary proliferation and differentiation, namely mammary-derived growth inhibitor and pleiotrophin (Kurtz), insulin-like growth factors and their interaction with oestrogen (Westley) and transcriptional activation by oestrogen receptors (Parker). A feature of these contributions is some informative schematics.

Section 2 relates to milk production, the ultimate end product of the fully differentiated mammary gland. Separate chapters consider physiological regulation of lactation by feedback inhibition, which occurs during milk storage within the gland (Peaker and Wilde), and molecular controls which influence milk protein secretion (Burgoyne) and gene expression (Rosen and Altiok/Groner).

Section 3 on Mammary Transgenics describes strategies for transgene expression in the lactating glands of rodent and farm animals to use for experimental purposes (Clark) and commercial exploitation (Coleman). The chapters which describe the effects of overexpressing genes such as c-*erbB2* and *TGF-α* in relation to mammary hyperplasia and neoplasia (Muller, Jolicoeur and Davies), lead logically into the second half of the book which concentrates on mammary cancer.

Section 4 on Genetic Changes in Mammary Cancers comprises chapters on (i) somatic abnormalities identified by cytogenetic and molecular analyses of primary breast cancers (Callahan, as a bonus, includes an account of how MMTV integration sites in a mouse mammary model system might identify genes involved in tumorigenesis), (ii) the role of overexpression of type 1 growth factor receptor family in malignant transformation in experimental systems (Cohen) and human tumours (Gullick) and (iii) the involvement of *BRCA1* and *BRCA2* genes in inherited predisposition to breast cancer (Ponder). Leinster is given the all-important task of demonstrating how molecular genetics have affected clinical management of breast cancer – while he appears to have found difficulties in practical examples of this, no-one would disagree with his conclusion that ‘the impact of molecular biology on clinical management is a revolution waiting to happen’.

Section 5 is the final section and appropriately is devoted to the final stage in cancer progression, i.e. Mechanisms of Mammary Metastasis. Chapters consider mortality genes (Nicolson), cell adhesion molecules (Alford/Taylor-Papadimitriou), Nm23 (Freije/Steeg), DNA transfer experiments (Barraclough) and matrix metalloproteinases (Cockett/Docherty).

While the meat of the book is the formal contributions from the symposium participants, it would be wrong not to mention the preface put together by Professor Rudland and his colleagues. This is an exquisite cameo summarizing the contents of the book and the logic behind its formulation.

A minor criticism is that, like other multiauthor texts, there are irritating inconsistencies in the degree of detail and style between chapters. For example, length of contributions varies between five and 17 pages, number of references range from 8 to 95 and some chapters have no experimental detail while others include extensive description of methodology (including what must be a world record long figure legend). This apart, the book represents an informative source of knowledge not only for specialists involved in mammary gland development and cancer but, because the gland is an excellent model for other basic processes such as differentiation, proliferation and transformation, also for cell and molecular biologists whose primary interest lies elsewhere. For my own part, I was delighted to have, as reward for preparing this review, the opportunity to place the volume on my bookshelf and use it as a reference text.

*Professor WR Miller,*
*Department of Clinical Oncology,*
*Western General Hospital,*
*Edinburgh*